# When are clients helpful? Capitalising on client involvement in professional service delivery

**DOI:** 10.1371/journal.pone.0280738

**Published:** 2023-02-22

**Authors:** Na Fu, Patrick C. Flood, Denise M. Rousseau, Tim Morris, Murray Johnstone

**Affiliations:** 1 Trinity Business School, Trinity College Dublin, The University of Dublin, Dublin, Ireland; 2 DCU Business School, Dublin City University, Dublin, Ireland; 3 Heinz College of Information Systems and Public Policy and the Tepper School of Business, Carnegie Mellon University, Pittsburgh, Pennsylvania, United States of America; 4 Saïd Business School, University of Oxford, Oxford, United Kingdom; 5 Sagulo Coaching & Mentoring Services Ltd, Glenlochar, United Kingdom; The Hong Kong Polytechnic University, HONG KONG

## Abstract

Professional service firms apply specialist knowledge to create customised solutions to client problems. In their work, teams of professionals undertake projects in which clients may be closely involved in co-creating solutions. However, we know little about the conditions under which client involvement contributes to better performance. We examine the direct and conditional contribution client involvement can make to project success and propose team bonding capital as a moderator. We conduct multi-level analysis of data from 58 project managers and 171 consultants nested in project teams. We find a positive impact of client involvement on both team performance and team member idea creativity. Team bonding capital moderates the relationships client involvement has with both team performance and individual member idea creativity, where the impact of client involvement is greater when team bonding capital is high. Implications for theory and practice are discussed.

## Introduction

The clients of professional service firms often get involved in the consulting process, helping to diagnose problems and customize solutions. Yet, client involvement is a mixed blessing. Clients can help onboard consultants, providing details to better diagnose the client’s problems. However, members of the client organization need not agree on problem definitions or support the same solutions. They can attempt to steer consultants toward a favoured diagnosis or preferred course of action, complicating their involvement in the consulting process. In the literature on service research, client involvement has mixed effects [1–5]. Existing theories of collaboration address service co-creation [6], value co-creation [7], client participation in service innovation [8], and design thinking in innovation [9]. All these studies posit that innovation is facilitated by client involvement in service design and delivery. However, client involvement does not always lead to positive outcomes. Clients may hinder innovation by adding complexity to service creation and delivery [10] or by politicizing the consulting process. Thus, a critical question to address is when does client involvement improve or hinder innovation, creativity, and performance in professional service work?

Client involvement refers to the degree clients participate in project-related work, taking the form of providing the consulting project team with knowledge, feedback and problem-solving skills. Limited research exists on client involvement in professional service firms (PSFs) such as consulting and law firms, being more focused on firm-level new product development [11, 12]. In the broader literature, client involvement is expected to have positive effects through firm-specific knowledge and insights they provide regarding the problem to be solved. However, validation of this proposition in the professional service context is needed.

In professional service contexts such as consulting, clients work closely with consulting project teams to diagnose and solve problems. Clients possess knowledge about the problem [5, 13] and can offer ideas and suggestions to service providers [14–16]. Clients also monitor [17, 18] and evaluate the quality of the service [19–21]. On the service provider side, many consulting firms adopt a process consulting approach where consulting teams engage in problem diagnosis, provide recommendations, and then participate in varying degrees of solution implementation [22, 23]. For the consultant seeking to maximise PSF earnings, collaboration with clients facilitates resource sharing and problem solving, accessing client specific-knowledge and with it the opportunity to build relationships that generate future work.

However, the benefits of such close relationships between clients and teams can come with costs for the PSF. As service providers, they can become dependent upon the client for efficient and effective project implementation. For the project to succeed, the client must provide staff with the time and skills to effectively engage with the consulting team. At the extreme, this dependence constitutes a form of ‘capture’ for the professional service firm, putting its reputation at risk if client supports are inadequate for project success. Although service co-creation can appear beneficial, it may not always lead to optimal outcomes.

The current study investigates the role of client involvement in the consulting team’s capacity to deliver quality service. It does so by examining both direct and boundary conditions for the performance impact of client involvement. Client involvement offers an important resource for service teams seeking deep knowledge about the business problems the client firm faces [24]. Informed by the extended team input, process, and performance model [25], which proposes that team process moderates the relationship between team input and output, we suggest that boundary conditions exist which influence the performance impact of client involvement. Using social capital theory [26–29], this study investigates a key quality of the service team itself, that is, relationships among team members. Relationships represent an aspect of team process, which may be expected to moderate the performance impact of client involvement. We examine the quality of team relationships using the concept of team bonding capital, that refers to a strong sense of belonging and desire to work in the team [25].

In the demanding context of professional service work, we investigate team bonding capital as a moderator of the impact of client involvement on project-related outcomes, specifically team performance and team member idea creativity. Team performance refers to the extent that the team completes projects of high quality and on time, a commonly used measure of team effectiveness [30]. To deliver professional services, consultants offer customised solutions that require innovative and feasible ideas from individual team members, itself another key indicator of project-based outcomes [8]. Team bonding capital can enhance the team’s capacity to deal with the many facets of client involvement, promoting cooperative interactions between team members and clients. In doing so, team bonding capital is expected to strengthen the impact of client involvement on team performance as well as the project contributions team members make in terms of both the novelty and quality of ideas. This study investigates the performance impact of client involvement and its interaction with team bonding capital on PSF team performance and individual member creativity.

The present study makes three contributions. First, it extends research on teamwork and collaboration in professional service organisations by focusing on the role of external clients. Second, it explores the conditions facilitating the performance impact of client involvement. In doing so, it addresses a priority in service management research [31], that is, how co-creation between service providers and users can enhance service quality. Third, it simultaneously addresses key drivers of consulting service delivery and internal team management as reflected in relations among project team members and external client management in the form of client involvement. Thus, it contributes to the professional service management literature by focusing on both internal and external factors to improve team effectiveness.

## Literature review and hypotheses development

### Client involvement in professional service organisations

Increasingly, organisations involve clients in new product development and service delivery. Scholars began to build theory regarding service organisations and the role of clients in service delivery four decades ago [5, 32]. A theoretical paper [32] noted that for clients to be effective as members of consulting teams they need the requisite skills and motivation and to be aligned appropriately to the team’s tasks. Clients may even come to be viewed as “partial” employees of service organisations, indicating their importance across stages of the service delivery process. Focusing on professional service teams, this study investigates the role of client involvement, that is, the degree of client participation in consulting project-related work.

Integrating client involvement into the service process has important consequences. Client involvement helps the service provider better understand project requirements and gather feedback on proposed solutions [33]. Clients are central to the work of process-oriented service firms where customised solutions are required [3, 34]. Among these firms, PSFs providing customised services need to work closely with their clients throughout the project. They typically co-produce services through interaction between the team’s professionals and its clients [1, 35, 36] with each participating in diagnosis, solution-generation and implementation. Indeed, service providers typically interact with clients before the service is contracted [10]. Clients can be viewed as co-creators by virtue of their feedback and help in generating solutions [37, 38]. The subsequent success of service delivery depends on these interactions remaining positive throughout the project [3, 39, 40]. Despite its importance, client involvement in consulting teams is poorly understood.

Management consulting is delivered by teams of professionals because the volume and complexity of its work typically cannot be performed by one person [36, 41]. Team output is co-produced with the client as in the case of training programs or organisational change activities [42]. Projects typically start with the partner receiving a client request. That partner then selects a project manager and team members with requisite expertise [16, 30]. The consulting project teams we studied provide relatively complex services with high customisation. Client involvement supports better project specification and execution. Consultants often work at client sites [43] to closely involve the client in the project, allowing consultants to take specific features of the setting into account, leading to project success [1, 44, 45].

### Client involvement and its outcomes

From a resource perspective [28, 46], client involvement constitutes an input: Clients provide teams with knowledge about the problem(s) and feedback on solutions. As a team input, following the classic input-process-output (IPO) model [24], client involvement typically is expected to lead to positive outcomes. Some studies have found positive effects for client involvement in the general service context. For example, in the banking industry where clients and service providers interact frequently, client involvement is found to be positively linked with perceived service performance [47]. In the context of the enterprise resource planning (ERP) systems implementation process, client involvement, in the form of knowledge sharing, provides both intrinsic and extrinsic motivation to the teams who provide ERP systems implementation [48]. In tourism service, some tourism service providers involve their clients to co-create knowledge in the service delivery [49].

Client involvement is associated with positive effects in a variety of other service environments. Patient involvement in healthcare treatment reported by professional clinical staff led to increases in staff job satisfaction [50]. Patient involvement also has been found to enhance interpersonal relationships between patients and clinical staff [51], facilitating clinical staff’s access to and learning from patients’ feedback through co-learning [52], and staff responsiveness to patients’ needs [53]. Relatedly in the telecom context, client involvement in the professional service design process led to the development of more original and valuable services [54].

In the professional consulting context, we too expect positive relationships between client involvement and project outcomes. Two outcomes investigated in this study are team performance and individual member idea creativity. Team performance is a common outcome variable in management research generally and in professional service teams [30, 55]. Performance of professional service teams includes completing tasks on time, meeting quality standards, and solving problems effectively. In addition, professionals need to come up with creative and feasible ideas to help clients solve their business problems [8]. New idea generation, when creative and feasible, helps in serving the client. Individual member idea creativity is used here as a dependent variable.

Client involvement can help deepen the consulting relationship and bind the client to the service team by promoting familiarity, attachment, and close working relationships [3]. Clients can share their expertise and knowledge with the team to help quickly diagnose problems. They can also offer alternative perspectives, helping the team to come up with creative and useful ideas. Developing deep understanding of client needs is an investment PSFs make in order to increase client switching costs and create long-term relationships, enhancing competitive advantage for both firms [56]. For example, when a PSF has long term relationships with their clients and deep understanding of its clients’ context and challenges, these clients are more likely to repeat their work with this service provider to avoid switching costs. Such commitment of PSFs to their clients reduces client perceptions of service uncertainty and ambiguity and their need for monitoring [3]. As a function of co-creation processes in consulting, we expect a positive direct effect of client involvement with both team performance and individual member idea creativity. Thus, we hypothesise:

Hypothesis 1. Client involvement will be positively related to team performance (1a) and individual member idea creativity (1b).

### Boundary conditions for the performance impact of client involvement

Despite its potential value, client involvement can be challenging. Client involvement or customer participation can be a major source of input uncertainty, due to differences in the quality both of this participation and the information clients provide to the consulting process [57]. Client involvement in service design and delivery can help to decrease both task uncertainty [e.g., how to make sense of client information) and workflow uncertainty [e.g., when to address client information needs) [58]. At the same time, involvement makes time and bandwidth demands on the consulting team. In financial services, client involvement increases employee job satisfaction, job performance and client satisfaction, but also contributes to employee job stress [10].

In the context of professional services, client understanding of the problem can vary [1, 5]. At the same time, many large client organisations have substantial in-house expertise and resources, providing ideas and insights to support their service providers. Further, the complex nature of the problems that professional service firms address typically requires some degree of client input to generate acceptable solutions. In turn, this close involvement offers clients some ability to monitor the service [32] and evaluate service quality in terms of process as well as outcomes. Close client involvement therefore has benefits but also increases transaction costs for service teams via frequent meetings and reviews and from demands for information overload. Thus, we need to understand when or under what conditions client involvement leads to higher team performance.

As laid out above, the traditional team IPO model [24] specifies that client involvement can be a valuable input for team performance and individual creativity. More recently, the IPO model has been extended to account for the more complex, dynamic, and adaptive team dynamics that apply to contemporary teams such as consulting teams [25]. Rather than a simple linear effect, where the team takes one step that then leads to another as in traditional manufacturing or office factory work, interactions are expected between inputs and team processes to account for outcomes [25]. In this regard, team process (e.g., social interactions among team members) can act as a boundary condition enhancing or impeding the effect of team input (e.g., client involvement) on outcomes.

Research has investigated some boundary conditions in the team input-outcomes relationship. For example, in research and development teams, team functional diversity, an input, does not always lead to knowledge sharing or innovation [59]. They found that team trust as a team process moderates the relationship between team diversity and team performance, such that team diversity increases knowledge sharing and team innovation when team trust is high [59]. Similarly, in a study of 88 highly interdependent production and assembly teams [60] serving diverse clients, team justice climate is regarded as an input and team justice climate strength is a team process variable. These input and process variables interacted to impact team performance and absenteeism [60]. Lastly, in top management teams research, team debates as a team process moderated the relationship of team diversity, a team input, with company performance [61].

Informed by the extended team IPO performance model [25] and the aforementioned research on the interaction between team input and process, we argue that benefits attained via client involvement are likely to be predicated on the existence of a highly functional team process that contributes to greater team member idea creativity and team performance. To identify potential conditional indicators, we consider relationships within consulting teams as critical for their learning, creativity, decision making and task completion. As noted above, client involvement may increase project complexity for teams by introducing competing points of view, alternative goals, and diverse interests. In interactions with clients by service project teams, intra-team factors are critical in the uptake and use of client knowledge [62]. A key factor in team effectiveness is the quality of relationships between team members [16, 55, 63, 64]. In consulting firms, it is common that team members will be chosen for similar future projects if they have previously worked well together [16]. This was confirmed by the senior HR partner in our study organisation who indicated that team members with positive experiences of working together tend to be chosen for repeat assignments. Strong relationships among team members are important for team success. Drawing on social capital theory [26, 27, 65, 66], the present study drills down into intra-team process and identifies an important relational moderator of the performance effects of client involvement: team bonding capital.

### The moderating role of team bonding capital

Team bonding capital “reflects affective feelings that team members hold toward each other and the team”, it “goes beyond trust and reflects a strong sense of rapport and a desire to stay together” [25] (page 526–527). Team bonding capital overlaps with other team-level process factors such as team cohesion [63]. Team cohesion is “the degree to which team members work together as they pursue the team’s goals” [67] (page 774). It includes both task integration (e.g., reaching the same goal) and social integration (e.g., attending social events together). Distinct from team cohesion, which involves identification with the team, team bonding capital represents social cognitions among team members pertaining to feeling close to each other and perceiving a sense of caring among members. Like the general notion of social capital [27], it encourages trust, knowledge exchange and idea combination in teams.

Team bonding capital is important in organisations using project-based teams [68] because their services are complex [15, 55, 69–71]. Team bonding capital has been found to motivate cooperative interactions in project-based teams [72] and along these lines, we posit that high team bonding capital promotes team motivation to acquire, share and integrate knowledge from different sources. We propose that team bonding capital enhances the relationship between client involvement and individual member idea creativity and team performance because highly bonded team members are motivated to collaborate and work together on collective goals.

From an agency perspective [73], clients are principals, and service providers are agents. For professional services, although clients may have at most, moderate knowledge about how to solve the problem [5, 74] they may still contribute valuable ideas and suggestions. Clients also have some ability to monitor the service [5] and will evaluate its quality [19]. This creates a type of agency model where clients represent the principal, and the service provider represents the agent. The challenge for the clients is to ensure that the agent’s (the PSF) interests are aligned with their own. When team bonding capital is low, agency costs to the service teams and client can be high. Knowledge sharing in weakly bonded teams is likely to be limited, undermining the generation of new knowledge even with high client input. Team members can find it difficult to present a united front to the client, further undermining team performance. Lack of knowledge sharing increases the costs for both clients and service teams since agreement on diagnosis and action takes more time. For service providers, when team members are closely bonded, knowledge tends to flow in a reciprocal fashion reducing ambiguity and conflict, leading to higher levels of performance [75, 76]. As a result of their attachment to each other, decisions can be made more quickly. Team bonding, thus, reduces transaction costs both within the team and with the client. In this way, team bonding capital interacts with client involvement and leads to the creation of more value for both service teams and clients themselves.

Team bonding capital, represented by close relationships, caring and being able to rely on each other, enhances collaboration, cooperation, and trust among team members [77, 78], helping them cope with work and client demands. When the level of client involvements is high, team bonding capital is expected to facilitate communication between the team and client, enable the team to quickly respond to the client’s feedback or suggestions and make more effective decisions on project-related matters. This, in turn, will promote collaboration, cooperation and trust, all of which have been found to enable consultants to work better with clients in the service co-creation process [79].

In the professional service context, when team bonding capital and client involvement are high, individual team members are more likely to better understand the client’s problem and generate creative and useful solutions. With these features in place, teams are more likely to coordinate and collaborate with clients, enabling client perspectives and concerns to inform solutions. When team bonding capital is low, team members are less motivated to share knowledge with each other, experience delays in the communication process, and take less responsibility for client requests. Such teams are likely to experience low efficiency, weak accountability, and negative outcomes. Therefore, we argue that in strongly bonded teams, client involvement is more likely to lead to positive outcomes including member idea creativity and team performance compared to weakly bonded teams.

Thus we propose the following hypotheses:

Hypothesis 2a. Team bonding capital will moderate the relationship between client involvement and team performance such that team performance will be higher when team bonding capital is higher.

Hypothesis 2b. Team bonding capital will moderate the relationship between client involvement and individual member idea creativity such that idea creativity will be higher when team bonding capital is higher.

**Fig 1:**
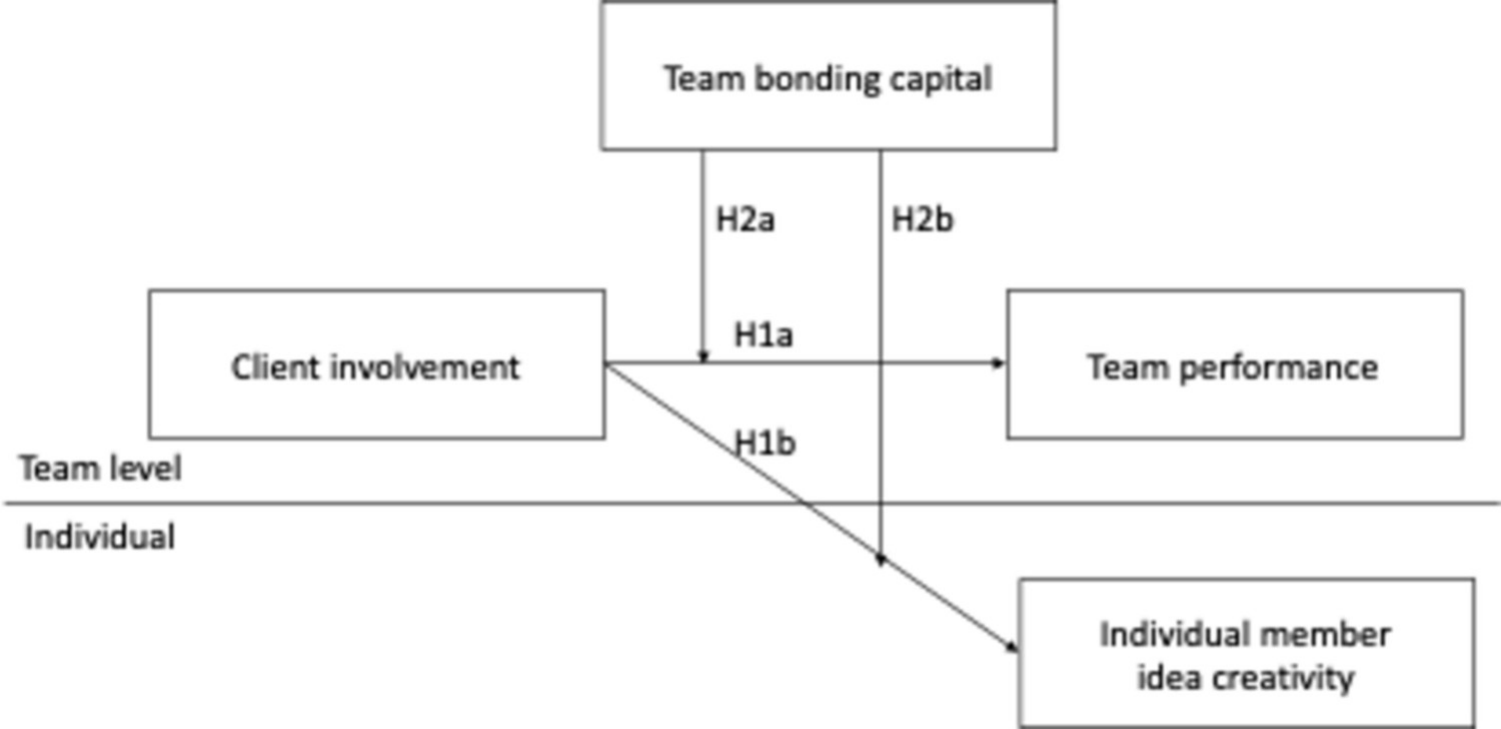
Research model.

Fig 1 presents the research model of this study.

## Methodology

### Data collection and sample profile

We studied PremierConsult (pseudonym), a global consulting firm employing 2,500 people across offices in North America, Europe, the Nordics, the Gulf and Asia Pacific. It services a wide range of industries including energy, financial services, life sciences and healthcare, manufacturing, government and public services, defence and security, telecommunications, transport and logistics. In our communications with the senior HR partner, we described the objectives of this study and requested access to project teams. In the professional service context, consultants typically charge clients on a time basis making consultants’ time very costly. The senior HR partner agreed to allow the teams to participate, providing access to 60 consulting teams. The average team size was 7, with a range from 4 to 16. Due to time constraints placed on consultants, we gained access to the project manager and 3 members per team. Participating team members were randomly selected. To ensure the effectiveness of our sampling strategy, we ran Analysis of Variance (ANOVA) and found no differences between the selected and non-selected members in terms of gender, age and tenure. Thus, we proceeded with data collection. A web-survey assessed their experience with team bonding capital. Client involvement and team performance were evaluated by team leaders.

As this study collected data from employees via an online survey, there were no risks envisaged for either researchers or participants during the data collection process. The survey questions were generic which in no way identified the participants. Participation was voluntary and participants could stop at any time. This project was deemed as a low-risk social research project under Notification Procedure at Dublin City University. A written ethical approval was granted from the Dublin City University Research Ethics Committee.

PremierConsult specialises in strategy implementation helping clients with organisational design and delivery, or what it refers to as “management augmentation.” Its services distinguish it from other well-known strategy consulting firms. All projects are highly tailored, albeit guided by PremierConsultant’s consulting process model. Project teams are made up of a mix of clients and PremierConsult professional staff. Having clients on teams is thought to be critical for the successful delivery of the projects both to solve the problems that clients face and to ensure mutual ownership of solutions as they are implemented. In PremierConsult, clients join the consulting teams in what the firm calls joint diagnosis, vision generation and plan development with joint responsibilities for service design and delivery. The information provided by clients facilitates relationship building, analysing, envisioning problems as well as planning and executing the projects. As the senior HR partner put it: *[PremierConsult] is not a methodology-driven consulting company*. *Essentially all work starts from the ground up to solve the problems the clients face*. *[During this process]*, *we bring some insights from previous clients’ work*. *But that would be helping to form hypotheses rather than forming methodology*. *The methodology that has been consistently used is the consulting process model*. *What we require working through various stages of the journey was the client to get the sense of engagement*”. PremierConsult emphasised that although it used its own distinctive consulting process, it did not do ‘cookie-cutter’ consulting: each project was treated as unique and not a repeat of a previous assignment.

A total of 233 responses were received, consisted of 60 project managers and 173 team members (response rate 97%). Removing incomplete responses, the sample size was 228 (95%) consisting of 171 team members (95%) and 58 team managers (97%). Among valid respondents, 78% were male, 97% were permanent staff, 34% held junior positions, 34% middle and 32% senior positions. Respondents averaged 6.46 years in the organisation (S.D. = 6.34).

### Measures

#### Team performance

Team performance was measured with a six-item team performance scale [80]. Ideally, it would be more objective to obtain such a rating from clients, but we did not have client access. Team performance thus was evaluated by project managers, as is common in management research [81, 82]. Project managers oversee task completion, scheduling service delivery and ensuring that completed tasks meet standards. Thus, they are well-positioned to rate the team’s performance. Managers indicated their extent of agreement on items using a five-point Likert scale (1 = strongly disagree, 5 = strongly agree). Example items included: “Our team completes its tasks on time”, “Our team makes sure that products and services meet or exceed quality standards”, “Our team responds quickly when problems come up” and “Our team successfully solves problems that slow down our work”. Alpha reliability was .87.

#### Individual member idea creativity

Idea creativity refers here to the innovativeness and feasibility of the ideas individual team members provide to the project. The feasibility and usefulness of ideas individuals propose to help solve client problems are critical to project success [8, 83]. Creativity is measured by the project manager’s evaluation of each team member. Three items were adapted from [84] to evaluate the novelty, workability and relevance of members’ ideas. Team members were evaluated on these statements: “This person comes up with ideas that are original,” “This person comes up with ideas that are workable (feasible),” and “This person comes up with ideas that are relevant and effective at solving the problem.” Alpha reliability was .81.

#### Client involvement

Client involvement was measured using six items [85]. In professional service firms, project managers typically interact with clients directly, seeking input to understand client needs and shape project delivery. As the focal point for many client interactions, they are appropriate raters. In this study, project managers assessed client involvement using a five-point Likert scale (1 = strongly disagree, 5 = strongly agree). Example items included: “Our clients often share their expertise and knowledge with our team”, “Our clients often provide our team with different perspectives and viewpoints” and “Our clients seldom offer information and alternatives for solving problems (reverse coded)”. Alpha reliability was .80.

#### Team bonding capital

Three items [78] were adopted to measured team bonding capital. To capture the quality of relationships among all members, both leaders and subordinates working in the team were asked to complete team bonding capital items. The referent is “team” and individual responses were aggregated to the team level. Respondents were asked to what extent they agreed with the following statements: “In my assignment team, I feel close to other team members at work”, and “In my assignment team, I feel a sense of caring for each other in my team at work”. We carried out t-tests comparing project managers and team members and found no difference between them on team bonding capital. Alpha reliability was .74.

Various techniques have been used to determine the appropriateness of aggregation. We used two aggregation tests: inter-rater agreement and inter-rater reliability [86]. Inter-rater agreement was assessed using *R*_*wg*_ [87, 88]. The mean of *R*_*wg*_ for team bonding capital was .90, well above the rule of thumb for *R*_*wg*_ of .60 [89] and the more commonly acceptable value of .70, indicating that team members and project managers agreed on team bonding capital at team level. Both inter-rater agreement and inter-rater reliability were assessed using intra-class correlations. ICC(1) is the amount of variance in the variable of interest that can be attributed to team membership. ICC(2) can be viewed as the reliability of the means. ICC(1) and ICC(2) were calculated with a one-way random-effects analysis of variance [90]. In our study, the ICC(1) value for team bonding capital was .15, within the recommended range of .05–.20 [91] and higher than the median value of .12 [89]. The ICC(2) value for team bonding capital was .40, lower than the .60 cut-off point [92] but comparable to coefficients in other studies [93]. The lower ICC(2) may be due to the small team sizes [94]. Based on these results, team bonding capital responses from team members and project managers were aggregated to the team level.

#### Control variables

We controlled for team size and percentage of female members which are potential influences on team dynamics and outcomes [30]. Team size is the total number of team members, a key factor in team member exchange and trust [95]. Percentage female was the number of female workers divided by team size, both provided by the organisation, an important factor in previous team studies [96–98]. Both team size and percentage of female members in the team were operationalised as a natural log. We controlled for gender, job grades, and tenure with the line manager. Gender was coded 1 = female, 0 = male. Grades were coded in the following rank order (1 = Analyst/Consultant Analyst/ Consultant, 2 = Principal Consultant, 3 = Managing Consultant, and 4 = Director/Partner/Senior Partner). Tenure with the line manager was assessed in months.

### Common method bias and analyses

Given that managers provided information on several variables, including client involvement, team bonding capital and team performance, common method bias (CMB) might be a concern. To address CMB, we followed several recommendations [99, 100]. For instance, before launching the survey, it was piloted with a group of managers and team members and revised and retested several times. Changes made as a result included question wording and order. In addition, we assured confidentiality to participants. We then assessed the common method variance by carrying out a series of CFA to establish the validity of the studied variables. The three-factor CFA model showed a good model fit (*χ*^*2*^*/df* = 124.35/100 = 1.24, *p* = .05; CFI = .94; RMSEA = .06; SRMR = .08). We then carried out *χ*^*2*^ difference tests that compared this model to three alternative nested models, as shown in Table 1. The comparison results reveal that the model fit of the three-factor model was significantly better than the alternative models (all at *p* < .001), suggesting that the variables evaluated by the managers were distinct, making CMB unlikely.

**Table 1:** Fit statistics from CFA comparison.

Models	*χ* ^*2*^ */df*	*CFI*	*RMSEA*	*SRMR*	*Δ*χ2	*Δdf*
**Three-factor model**	**124.35/100**	**.94**	**.07**	**.07**		
Model A^a^	197.52/102	.77	.13	.10	73.17***	2
Model B^b^	174.83/102	.82	.11	.12	50.48***	3
Model C^c^ (Harman’s Single Factor Test)	305.33/107	.52	.18	.23	181.09***	7

Notes: N = 59

***p < .001; *χ^2^* = chi-square discrepancy, *df* = degrees of freedom; CFI = Comparative Fit Index; RMSEA = Root Mean Square Error of Approximation; SRMR = Standardized Root Mean Square Residual; *Δ*χ2 = difference in chi-square, *Δdf* = difference in degrees of freedom. All models are compared to the three-factor model.

^a^ = Bonding capital and team performance combined into a single factor.

^b^ = Bonding capital and client involvement into a single factor.

^c^ = All factors combined into a single factor.

To test hypotheses related to team performance (H1a and H2a), hierarchical multiple regression analysis [101] was conducted. Moderation was tested using the moderated regression analysis [101]. All variables were standardised to avoid multicollinearity. For testing hypotheses related to individual member idea creativity (H1b and H2b], cross-level moderation analysis [102] was used in Mplus since the model involves both team (client involvement and team bonding capital) and individual (member idea creativity) level constructs. In addition, we examined the extent to which member idea creativity varied between teams. The ICC(1) value for member idea creativity is .41. There was significant team-level variance in member idea creativity, which means team-level client involvement and team bonding capital could explain between-team variance in member idea creativity. In the analysis, we used grand mean-centering [102] for team-level variables (i.e., client involvement, team bonding capital and team-level controls) and group mean-centering [102] for individual-level variables (i.e., individual control variables), treating the interaction at the between level.

## Results

Table 2 shows descriptive statistics, including means, standard deviations, correlations, and reliabilities. Table 3 presents regressions testing Hypotheses 1 and 2.

**Table 2:** Descriptive statistics.

Variables	Mean	SD	1	2	3	4	5	6	7	8	9
*Team level*											
1. Team performance	4.33	.48	(.87)								
2. Client involvement	3.96	.47	.38**	(.80)							
3. Team bonding capital	3.99	.35	.43**	.02	(.74)						
4. Team size (log)	1.96	.52	.17	.17	.08						
5. Percentage of female members (%)	.26	.18	.01	-.21	.07	-.07					
*Individual level*											
6. Individual member idea creativity	3.92	0.67	.29**	.24**	.05	.03	-.10	(.81)			
7. Bonding capital	4.00	.53	.16*	-.13	.55**	.05	.08	.03			
8. Gender	0.27	0.44	-.10	-.16*	-.13	-.10	.38**	-.08	.01		
9. Grade	1.73	0.78	-.05	-.07	-.01	-.07	-.12	.29**	-.02	-.15*	
10. Tenure with manager	10.53	10.67	.11	.05	.12	.12	.03	.19*	.19*	-.11	.24**

Note: The numbers in the brackets are the Cronbach’s Alphas

** *p <* .01

* *p <* .05 (two-tailed test). The Level 2 variables were disaggregated before calculating within-level correlations. N = 155–171 at individual level; = 58–60 at team level.

**Table 3:** Results of regression.

	Team Performance			Individual Member Idea Creativity		
Variables	Model 1	Model 2	Model 3	Model 4	Model 5	Model 6
*Team Level*						
Team size	.17	.09	.09	.06	-.01	.04
Percentage of female members (%)	-.02	.08	.03	-.25	-.01	-.07
Client involvement		.38**	.32*		.35*	.20
Team bonding capital			.40***			.04
Client involvement * team bonding capital			.24*			.33*
*Individual Level*						
Gender				.03	.03	.04
Grades				.20*	.22*	.08
Tenure with manager				.24*	.22*	.22*
*R* ^*2*^	.01	.12	.32	.01	.12	.20

Note: N = 58 at team level and 154 at individual level (Listwise). Standardized coefficients were reported

*** *p* < .001

** *p <* .01

* *p <* .05

Hypothesis 1 proposed that client involvement is positively related to team performance (1a) and member idea creativity (1b). Results in Table 2 (Models 2 and 4) show that client involvement was positively related to team performance (*β* = .38, *p* < .01) and member idea creativity (*β* = .35, *p* < .05), supporting Hypothesis 1a and 1b.

**Fig 2:**
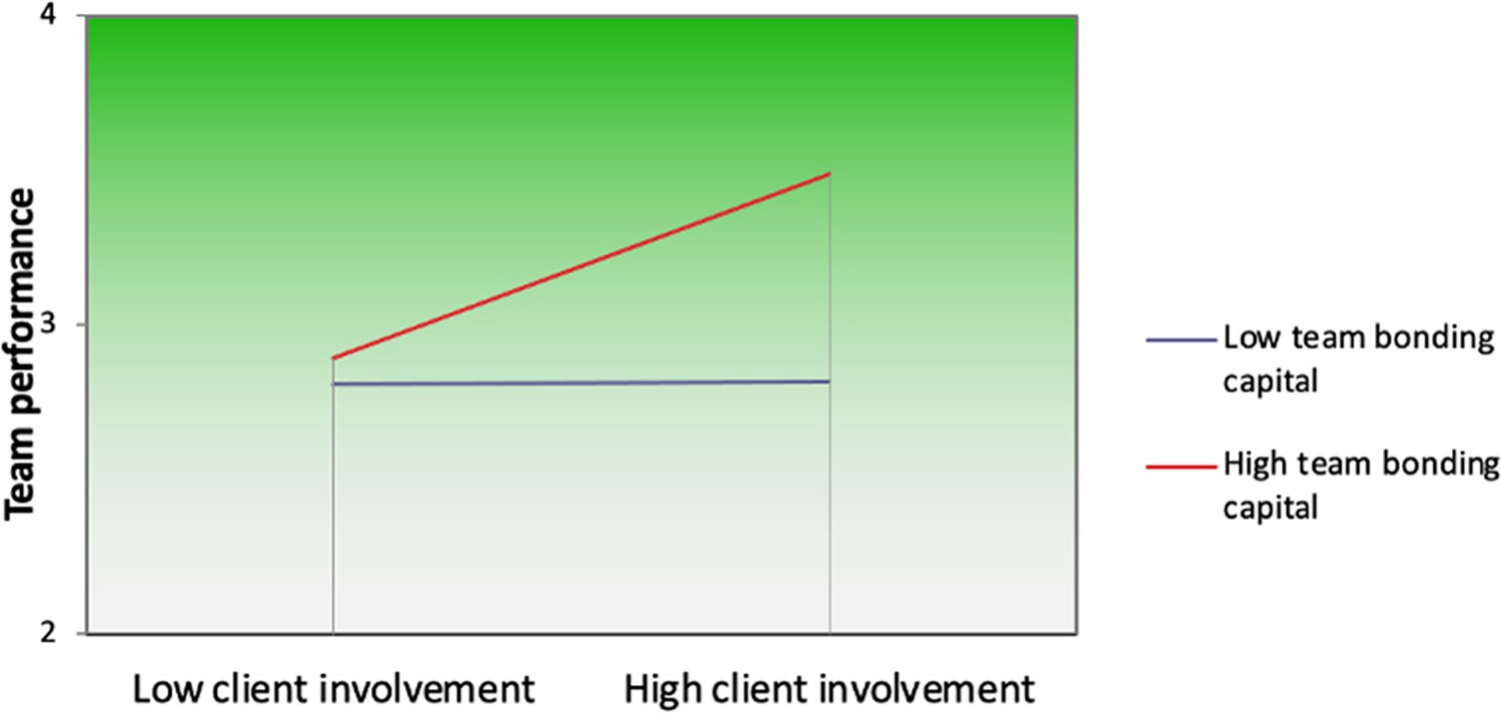
Plot for the interaction between client involvement and team bonding capital on team performance.

**Fig 3:**
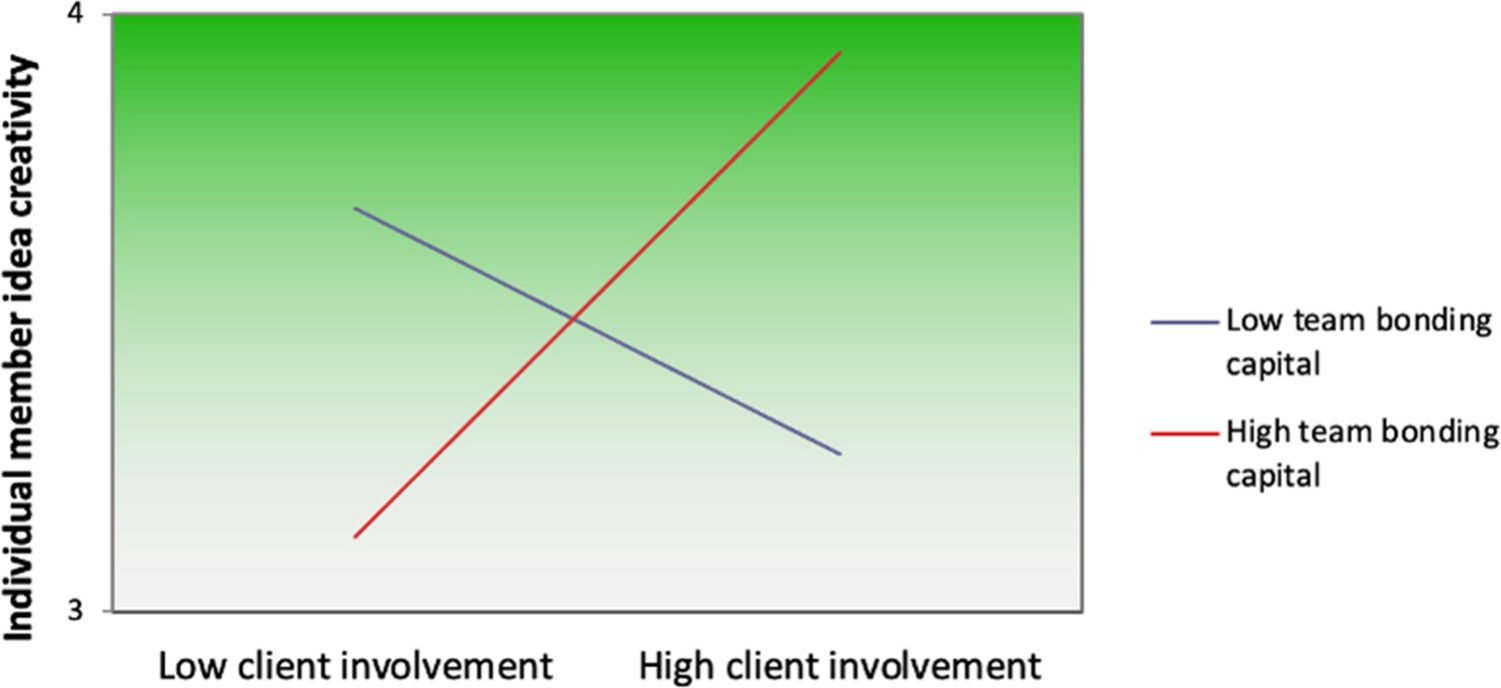
Plot for the interaction between client involvement and team bonding capital on individual member idea creativity.

Hypothesis 2 proposed that team bonding capital moderated the relationship of client involvement with team performance. Results in Table 2 (Model 3 and Model 6) show that interaction is positive for both team performance (*β* = .24, *p* < .05) and member idea creativity (*β* = .33, *p* < .05). Though it was not hypothesised, we observed a positive direct effect of team bonding capital on team performance (*β* = .40, *p* < .001). The interaction coefficients were significant for each outcome, supporting moderation. To illustrate the difference between high and low team bonding capital, Figs 2 and 3 plot interactions for both outcomes.

As shown in Fig 2, compared to low team bonding capital, the link between client involvement and team performance is stronger at high levels of team bonding capital. In other words, team performance increases with client involvement when team bonding capital is high. Team performance does not increase with client involvement when team bonding capital is low. This suggests that team bonding capital helps strengthen the link between client involvement and team performance. The simple slope test provides further support: The relationship between client involvement and team performance was positive and differed from 0 at high (*B* = .30, *T* = 3.74, *p* < .001) but not at low (*B* = .00, *T* = .03, *n*.*s*.) team bonding capital.

As shown in Fig 3, the relationship between client involvement and creativity was positive at high levels of team bonding capital (*B* = 1.05, *T* = 12.58, *p* < .001) and negative at low (*B* = -1.02, *T* = -10.11, *p* < .001). Thus, client involvement differs in its relationship with member idea creativity depending on the level of team bonding capital. Therefore, Hypothesis 2 was supported.

## Discussion

This study sought to better understand the phenomenon of client involvement during professional service delivery. Drawing on the classic and extended models of team input, process, and output [24, 25] and research on professional service teams, we found that client involvement played a role in improving team performance and individual idea creativity. Additionally, team bonding capital moderated its relationship with these outcomes. Client involvement leads to better outcomes when team bonding capital is high. When team bonding capital is low, client involvement does not affect team performance but does inhibit individual idea creativity. These findings inform our understanding of the conditions that can enhance the value attained from client involvement in professional service delivery. We now turn to the implications of our findings for scholarship and practice.

### Scholarly implications

Our findings address an important service research priority [31]; how to optimise the co-creation of services by clients and service providers. Integrating clients into the service creation process has been found to offer benefits in creativity and knowledge transfer in settings as diverse as the ERP implementation process [48], knowledge cocreation in tourism service [49], and hair stylist creativity [85]. By investigating the knowledge intensive and creative work performed in management consulting teams, we find that client involvement facilitates both project success and team member idea creativity, an effect influenced by the quality of the relations among consulting team members. These findings shed light on previous findings where client involvement was disruptive to the team or poorly managed by its members, highlighting the contributions of high quality within-team relations to optimising client involvement.

We suggest that co-creation is enabled when the consulting team itself has the capacity to respond appropriately to client input and integrate client involvement into its work. The team processes required to do so are likely to be related to the team’s capacity for reflection and honest conversation, capabilities enabled by team bonding capital. High team bonding capital enhances the team’s ability to make sense of client knowledge and concerns and adapt its own professional knowledge to the client’s context. Conversely, when teams have weak relationships, they are less able to make sense of input from the client and have difficulty accessing and adapting the knowledge team members possess.

Despite recognition of the centrality of co-production to services in PSFs [3, 8, 20, 74, 103, 104], little research exists on the phenomenon of client involvement in the context of consulting services or the conditions that support it. One reason is the fundamental assumption that client involvement broadly is positively related to individual and team outcomes. Another reason is the narrow focus of previous PSF team research on internal team composition [30] and team competence [105]. The present study extends our understanding of PSF team performance by demonstrating the role of team dynamics. These findings are made possible by our study’s quantitative design using multi-source data and multi-level modelling simultaneously investigating the consulting team and the individual member effects of client involvement. Prior research on integrating clients into the service co-design process relied either on qualitative methods or focused on the dyadic relationship between service provider and client [51, 106].

Importantly, this study contributes to a multidisciplinary perspective on the client value co-creation process. By identifying the intra-team factor of team bonding capital, it extends service research on client involvement beyond marketing to human resource management and organisational behavior. We note that Frey and colleagues [107] described how the co-interaction of clients and staff in PSFs is beneficial to staff satisfaction and retention. Client involvement is a key aspect of this interaction. Co-creating value with clients is a phenomenon of interdisciplinary interest, not merely a function of marketing or service development. It requires collaboration to develop a strong service climate so that individuals and teams can better interact with their customers and clients. We found that client involvement led team members to increase the usefulness and novelty of their ideas and solutions at the individual level. At the team level, client involvement helps to improve the service team performance. In this PSF, the emphasis on building lock step relationships with clients differs substantially from the off the shelf consulting packages some firms offer. PSF staff are embedded in the client firm as part of a mixed team while also formally constituting the service provider, a form of exocentric or externally oriented team that is more complex than traditional teams [108]. Continuing to drill down into the mechanisms operating in such boundary spanning teams can generate new team theory in future.

We suggest that future research look at the facilitators and barriers to team bonding capital and how it affects the discrete activities characterizing how the team processes inputs from clients. These activities may include how the team interprets client feedback or integrates diverse ideas into a coherent project plan. We posit that the service co-creation process requires absorptive capacity on the part of the team to deal with the diverse inputs collaboration generates [109], a capacity to which team bonding capital contributes.

Another important area for future research is how clients themselves can promote more effective relationships with consulting teams. The recognition gained from working with leading professional service providers has been shown to increase a client firm’s own market value [110]. Our findings indicate that client involvement is important to project success though no research yet exists from the client point of view regarding the opportunities and challenges these firms face in working with consultants. Time demands and personnel shortages can make it difficult to effectively work with consultants, particularly on projects requiring considerable co-creation. Large investments of time and people may be onerous for client firms, particularly when high-value-adding individuals are involved. Research is needed on client-side facilitators and barriers to effective consultant engagement.

### Practical implications

Client involvement contributes to the usefulness of ideas that consultants propose and the consulting team’s effectiveness. Our findings support adoption of a client active paradigm [31], in which PSFs engage with existing and future clients carefully and often. We recommend that managers and members who interact with their clients, regard clients or clients-to-be not as passive responders but as co-creators in the service provision process. We advise PSFs to design and deliver specific training programmes involving role plays, experience sharing and communication with clients, to increase their staff’s awareness of clients as co-creators. Doing so will help firms to benefit from engaging their clients at very early stages as well as coordinating and collaborating throughout service delivery.

A key condition supporting client involvement and effective co-creation is the bond among consulting team members. PSFs need to support quality team member relationships to increase their capacity for collaboration. Strongly bonded teams are better able to coordinate and communicate, which facilitates their ability to make sense of diverse client inputs in the service process. This finding has a clear implication for managers in PSFs. Before engaging with clients, managers need to ensure that bonding relations exist among team members, indicated by high levels of trust and sense of belonging. Managers could conduct 1:1 meetings with team members or check employee engagement survey results, if available, to assess the bonding levels among members. If levels are low, we recommend that managers intervene to develop and enhance bonds among team members, including organising social events or team building activities and creating open communication channels.

### Limitations and future research

Despite the contributions this study has made, a few limitations need to be noted. First, we studied a medium-sized consulting firm, raising issues of generalisability. The research context is a professional service firm providing customised solutions to client problems. Clients of such firms are typically large organisations with their own internal expertise and resources, giving them capacities for involvement that may differ from other clients (e.g., members of the public, patients, or employees in small firms). Relationships between service providers and clients are central to PSFs and frequently exist over time. The generalisability of our findings in other contexts thus warrants attention. We suggest investigating general service sectors and product industries where client interactions with providers are less regular and more short-term. National cultural differences also are known to affect client expectations, experience and trust thereby posing challenges for PSF teams [111]. Future research should evaluate our findings in other PSF contexts with a global scope.

Second, this study is limited in the methods used. The cross-sectional design does not permit testing causality. Although common method bias is unlikely, we encourage future research assessing independent and dependent variables at different times. Another limitation was our use of the observed-mean approach, using aggregated scores of team bonding capital. Our theoretical framework and the very high response rate supported the use of such approach, consistent with other multi-level studies [112–117]. Nonetheless, future research with cross-level interaction models is encouraged to use multilevel structural equation modelling [118].

Third, this study did not include the client point of view due to lack of access. However, we suggest that client involvement in study design and assessment can add value to research on effects associated with client involvement in the provision of PSF services. Thus, we suggest that future research incorporates client perspectives. We also suggest that additional moderators be tested, including the nature of the service provided from the perspective of the client, its perceived usefulness to the clients involved, variation in project-related client knowledge, the client experience of treatment by the service team, and the bonding between client and service team.

Related to the limitations of our outcome measures is the need to address effects at different phases in the consulting process. Such effects include how client involvement operates across project phases—from the buying process (the lead, opportunity, proposal and sale stages) to service implementation (joint diagnosis, vision generation, plan development and delivery). Focusing on consulting *phases* in studying client involvement can lead to more comprehensive understanding of how and when involvement helps create value.

Last, we suggest more attention to the antecedents of client involvement including why and when clients are more likely to be involved in the service design and delivery. Project priorities as perceived by the client is a likely antecedent. Other factors that influence client involvement may include perceived mutual interest, having relevant knowledge, developing positive psychological contracts, and creation of rapport with the service team, all of which can be beneficial to service delivery. Negative factors that warrant investigation include lack of clear incentives for client involvement, limited capabilities on the part of client staff, and time constraints. Overall, the antecedents of client involvement are likely to be important to understanding service co-creation.

## Conclusion

Teamwork and collaboration are important drivers of PSF service delivery. This study sheds light on how to capture benefits from involving clients in professional service delivery. Building quality relations among consulting team members can be critical to optimising client involvement and its impact on team member idea creativity and team performance. This study offers a starting point for better understanding how professional service firms can better collaborate with their clients via building their consulting teams’ internal team bonding capital.

## Supporting information

S1 Appendix (DOCX)
