# Supplementary material for: When are clients helpful? Capitalising on client involvement in professional service delivery
Source: PLoS One. 2023 Feb 22;18(2):e0280738. doi: 10.1371/journal.pone.0280738 (PMC9946238; doi:10.1371/journal.pone.0280738)
Supplement: S1 Appendix — (DOCX) [file pone.0280738.s001.docx]

**Appendix: Measurement Scales**

**Team performance**(83)

1. Our team meets or exceeds it goals.
2. Our team completes its tasks on time.
3. Our team makes sure that products and services meet or exceed quality standards.
4. Our team responds quickly when problems come up.
5. Our team is a productive team.
6. Our team successfully solves problems that slow down their work.

**Individual member idea creativity**(87)

1. This person comes up with ideas that are original.
2. This person comes up with ideas that are workable (feasible).
3. This person comes up with ideas that are relevant and effective at solving the problem.

**Client involvement**(88)

1. Our clients often share their expertise and knowledge with our team.
2. Our clients often provide our team with different perspectives and viewpoints.
3. Our clients do not help our team with hints or cues for new ideas (r).
4. Our clients seldom offer information and alternatives for solving problems (r).
5. Our clients often contribute new ideas about how to solve problems.
6. Our clients provide our team with constructive suggestions about the project

**Team bonding capital**(81)

1. In my assignment team, I felt close to my colleagues at work.
2. In my assignment team, I could count on my colleagues at work.
3. In my assignment team, I felt a sense of caring for each other at work.
